# Supplementary material for: Measuring Attitudes Toward Plastics: A Cross-Cultural Adaptation and Patient Evaluation Study
Source: Int J Environ Res Public Health. 2025 Dec 12;22(12):1857. doi: 10.3390/ijerph22121857 (PMC12732577; doi:10.3390/ijerph22121857)
Supplement: Supplementary file 1 [file ijerph-22-01857-s001.zip › Supplementary Methods 1.pdf]

Supplementary Methods S1. Sample composition planning phase for tool pretest

The pretest is the final stage of the adaptation process. This field test of the new questionnaire uses the prefinal version with subjects from the target setting (30 to 40 participants are recommended). It is therefore essential that respondents at this stage share the key characteristics of the population that may ultimately be included in the large-scale survey.

The composition of the sample was therefore planned taking into account the distribution by age, education level, and sex of the Italian population.

For this purpose, data from the permanent population census conducted by the National Institute of Statistics (Istat) for the year 2023 were employed. Specifically, the distribution by age and education level shown in Table 1 was initially used.

Table 1. Percentage composition by age and education level of the Italian population (No.48,238,000)

	Middle school or lower	High school	University degree
20-29 years	3,3	7,6	2,2
30-49 years	15,0	15,5	6,6
50-64 years	13,7	7,5	2,8
65-74 years	10,1	2,1	0,8
75 years and over	11,1	1,1	0,5

Since the data provided by Istat include the 15-19 age class, only the population starting from the next age class, i.e., 20 years and over, was selected, so as not to include minors.

Once this frequency distribution for the two variables had been obtained, the total number planned for the pretest (40 cases) was allocated according to the percentage values in Table 1, thus yielding the proportions shown in Table 2.

Table 2. Sample distribution (No.40) by age and education level based on the distribution in the Italian population (Table 1)

	Middle school or lower	High school	University degree
20-29 years	1	3	1
30-49 years	6	6	3
50-64 years	6	3	1
65-74 years	4	1	0
75 years and over	5	0	0

These quotas were subsequently further broken down by gender. In addition, rather than strictly maintaining perfect representativeness with respect to the Italian population, it was decided to over-represent the less educated groups, expected to have greater difficulty in understanding and completing the questionnaire.

Table 3. Sample distribution (No. 40) by age, education level and sex based on the distribution in the Italian population (table 1)

	Males			Females		
	Middle school or lower	High school	University Degree	Middle school or lower	High school	University Degree
20-29 years	1	1	0	1	1	0
30-49 years	3	2	1	3	2	1
50-64 years	3	2	1	3	2	1
65-74 years	2	0	0	3	1	0
75 years and over	3	0	0	3	0	0

It was also decided to involve at least two foreign citizens in the pre-test, considering that the foreign component of the population residing in Italy amounts to 10%.
