# Supplementary material for: Measuring Attitudes Toward Plastics: A Cross-Cultural Adaptation and Patient Evaluation Study
Source: Int J Environ Res Public Health. 2025 Dec 12;22(12):1857. doi: 10.3390/ijerph22121857 (PMC12732577; doi:10.3390/ijerph22121857)
Supplement: Supplementary file 1 [file ijerph-22-01857-s001.zip › Table S1_Clarity ratings per item.pdf]

Supplementary Table S1. Participant clarity ratings per item, *n (valid %).*

Item number	Very clear	Needs some changes	Not clear at all	No response
D1	43 (100.0)	0	0	0
D2	39 (92.8)	2 (4.8)	1 (2.4)	1
D3	41 (97.6)	1 (2.4)	0	1
D4	41 (97.6)	0	1 (2.4)	1
D5	41 (95.3)	2 (4.7)	0	0
D6	43 (100.0)	0	0	0
D7	31 (73.8)	11 (26.2)	0	1
D8	41 (97.6)	1 (2.4)	0	1
D9	30 (69.8)	9 (20.9)	4 (9.3)	0
D10	35 (83.3)	7 (16.7)	0	1
D11	40 (97.6)	1 (2.4)	0	2
D12	32 (86.5)	5 (13.5)	0	6
D13	36 (85.7)	6 (14.3)	0	1
D14	28 (70.0)	11 (27.5)	1 (2.5)	3
D15	38 (92.7)	2 (4.9)	1 (2.4)	2
D16	41 (100.0)	0	0	2
D17	35 (83.3)	7 (16.7)	0	1
D18	36 (87.8)	5 (12.2)	0	2
D19	39 (100.0)	0	0	4
D20	39 (95.1)	2 (4.9)	0	2
D21	40 (97.5)	1 (2.4)	0	2
