# Supplementary material for: Measuring Attitudes Toward Plastics: A Cross-Cultural Adaptation and Patient Evaluation Study
Source: Int J Environ Res Public Health. 2025 Dec 12;22(12):1857. doi: 10.3390/ijerph22121857 (PMC12732577; doi:10.3390/ijerph22121857)
Supplement: Supplementary file 1 [file ijerph-22-01857-s001.zip › Table S2_Questionnaire modified after citizen input.pdf]

Supplementary Table S2 – Questionnaire modified after citizen input

Item number	Item submitted to citizens	Final item
D1	What is the first word/phrase that comes to your mind when you hear the word "plastic"?	What is the first word/phrase that comes to your mind when you hear the word "plastic"?
D2	What is the first word/phrase that comes to your mind when you think about the positive effects of plastic use?	**What is the first word/phrase that comes to your mind when you think about the positive impact of plastic use?**
D3	What is the first word/phrase that comes to your mind when you think about the negative effects of plastic use?	**What is the first word/phrase that comes to your mind when you think about the negative impact of plastic use?**
D4	Select the three categories of products you most strongly associate with plastics.<br>• Cars<br>• Agriculture<br>• Construction and building products<br>• Clothing<br>• Electronics<br>• Food packaging<br>• All other non-food packaging<br>• Furniture<br>• Household items<br>• Medical products<br>• Single-use bags	Select the three categories of products you most strongly associate with plastics.<br>• Cars<br>• Agriculture<br>• Construction and building products<br>• Clothing<br>• Electronics<br>• Food packaging<br>• All other non-food packaging<br>• Furniture<br>• Household items<br>• Medical products<br>• Single-use bags
D5	We now present a list of nine environmental issues considered globally relevant. In your opinion, how serious is each of them?<br>• Air pollution<br>• Water pollution<br>• Amount of plastic waste produced<br>• Plastic in the sea<br>• Amount of general waste not recycled<br>• Climate change (global warming)<br>• Natural resource depletion (forests, water, energy)<br>• Endangered species and biodiversity	We now present a list of nine environmental issues considered globally relevant. In your opinion, how serious is each of them?<br>• Air pollution<br>• Water pollution<br>• Amount of plastic waste produced<br>• Plastic in the sea<br>• Amount of general waste not recycled<br>• Climate change (global warming)<br>• Natural resource depletion (forests, water, energy)<br>• Endangered species and biodiversity

Supplementary Table S2 – Questionnaire modified after citizen input

Item number	Item submitted to citizens	Final item
	• Water shortages	• Water shortages
D6	How concerned are you about each of the following issues?<br>• Plastic pollution in the sea<br>• Amount of plastic waste produced daily in Italy<br>• Non-recycled plastic	How concerned are you about each of the following issues?<br>• Plastic pollution in the sea<br>• Amount of plastic waste produced daily in Italy<br>• Non-recycled plastic
D7	Do you think the use of plastic packaging for food products is…<br>• Harmful/beneficial<br>• Bad/Good<br>• Inconvenient/Convenient<br>• Not useful/Useful<br>• Reduces/Increases food waste<br>• Makes my life harder/makes my life easier<br>• Reduces/Increases food hygiene<br>• Negative/Positive for the environment<br>• Negative/Positive for my health	**Do you think the use of plastic packaging for food products is…**<br>• **Inconvenient/Convenient**<br>• **Not useful/Useful**<br>• **Reduces/Increases food waste**<br>• **Makes my life harder/makes my life easier**<br>• **Reduces/Increases food hygiene**<br>• **Negative/Positive for the environment**<br>• **Negative/Positive for my health**
D8	How often do you perform each of the following actions?<br>• Reduce the use of “on-the-go” plastic (e.g., bringing my own water bottle or take-away container)<br>• Reduce the use of packaging (e.g., buying loose products)<br>• Reduce the use of single-use plastic (e.g., replacing plastic containers with glass, buy wooden household goods as opposed to plastic goods)	**How often do you perform each of the following actions?**<br>• **Reduce the use of “on-the-go” plastic (e.g., bringing my own shopping bag)**<br>• **Reduce the use of packaging (e.g., buying loose products or refills)**<br>• **Reduce the use of single-use plastic (e.g., replacing plastic bottles with a water bottle)**
D9	Rate the use of durable plastic products (containers, furniture, stationary) according to the following criteria:<br>• Harmful/Beneficial<br>• Bad/Good<br>• Inconvenient/Convenient<br>• Not useful/Useful	**Rate the use of durable plastic products (chairs, tables, plastic shelves, stationary) according to the following criteria:**<br>• **Inconvenient/Convenient**<br>• **Not useful/Useful**<br>• **Makes my life harder/Makes my life easier**

Supplementary Table S2 – Questionnaire modified after citizen input

Item number	Item submitted to citizens	Final item
	• Makes my life harder/Makes my life easier<br>• Negative/Positive for the environment<br>• Negative/Positive for my health	• **Negative/Positive for the environment**<br>• **Negative/Positive for my health**
D10	How much do you agree with the following statements?<br>• I would like to use less disposable plastic<br>• I would like to use less plastic for durable products (by buying items made with alternative materials)<br>• I have no control over how much disposable plastic I use	**In order to reduce plastic pollution...**<br>• **I would be willing to use less disposable plastic**<br>• **I would be willing to use less plastic for durable products (by buying items made with alternative materials)**<br>• **I believe my behavior can make a difference**
D11	How much do you agree with the following statements?<br>• These days, too many items are made out of plastic<br>• If all plastic is recycled, there is no need to reduce my use of it<br>• If plastic food packaging reduces food wastage, that justifies its increased use	How much do you agree with the following statements?<br>• These days, too many items are made out of plastic<br>• If all plastic is recycled, there is no need to reduce my use of it<br>• If plastic food packaging reduces food wastage, that justifies its increased use
D12	What is the first word/phrase that comes to your mind when you hear the word "bioplastic"?	What is the first word/phrase that comes to your mind when you hear the word "bioplastic"?
D13	Rate the use of bioplastic packaging for food products according to the following criteria.<br>• Harmful/Beneficial<br>• Bad/Good<br>• Inconvenient/Convenient<br>• Not useful/Useful<br>• Increases/Reduces food waste<br>• Negative/Positive for the environment<br>• Negative/Positive for my health	**Rate the use of bioplastic packaging for food products according to the following criteria.**<br>• **Inconvenient/Convenient**<br>• **Not useful/Useful**<br>• **Increases/Reduces food waste**<br>• **Negative/Positive for the environment**<br>• **Negative/Positive for my health**
D14	Do you agree or disagree with the following statements?<br>• All bioplastics are biodegradable<br>• I have used an item made from a bioplastic before	**Do you agree or disagree with the following statements?**<br>• **All bioplastics are biodegradable**<br>• **Items made from a bioplastic are increasingly**

Supplementary Table S2 – Questionnaire modified after citizen input

Item number	Item submitted to citizens	Final item
	• Some bioplastics are indistinguishable from conventional plastics	**common**<br>• **Some bioplastics are indistinguishable from conventional plastics**
D15	Considering packaging and food bags, do you think the following materials are better or worse for the environment compared to conventional plastic?<br>• Biodegradable plastic<br>• Biobased plastic<br>• Paper<br>• Degradable plastic<br>• Glass	Considering packaging and food bags, do you think the following materials are better or worse for the environment compared to conventional plastic?<br>• Biodegradable plastic<br>• Biobased plastic<br>• Paper<br>• Degradable plastic<br>• Glass
D16	Do you think biodegradable plastics are better or worse for the environment compared to recyclable plastics?	Do you think biodegradable plastics are better or worse for the environment compared to recyclable plastics?
D17	How much do you agree with the following statements?<br>• All plastics made from plants are biodegradable<br>• Leaving a biodegradable plastic food package at the beach shouldn't be considered as littering because the material is biodegradable<br>• I would worry less about plastic in the sea if it was biodegradable<br>• I would like more of the plastic items I use to be biodegradable<br>• Biodegradable plastics can have a negative impact on the environment<br>• I would be willing to pay up to 10% more if a product was made with biodegradable or biobased plastic instead of conventional plastic<br>• I would be willing to pay up to 10% more if a product was made with recycled plastic instead of new plastic	How much do you agree with the following statements?<br>• All plastics made from plants are biodegradable<br>• Leaving a biodegradable plastic food package at the beach shouldn't be considered as littering because the material is biodegradable<br>• I would worry less about plastic in the sea if it was biodegradable<br>• I would like more of the plastic items I use to be biodegradable<br>• Biodegradable plastics can have a negative impact on the environment<br>• I would be willing to pay up to 10% more if a product was made with biodegradable or biobased plastic instead of conventional plastic<br>• I would be willing to pay up to 10% more if a product was made with recycled plastic instead of new plastic
D18	At present, how would you dispose of a biodegradable plastic	**At present, how would you dispose of a biodegradable**

Supplementary Table S2 – Questionnaire modified after citizen input

Item number	Item submitted to citizens	Final item
	material (e.g., a food package or a take-away container)?<br>• Recycling bin<br>• Regular bin<br>• Bin for organic waste / food waste bin<br>• I don't know<br>• Other	**plastic material (e.g., a food package or a take-away container)?**<br>• **Recycling bin (plastics, paper, ecc.)**<br>• **Regular bin**<br>• **Bin for organic waste / food waste bin**<br>• **I don't know**<br>• **Other**
D19	In the last year, approximately what percentage of plastic bottles/containers has your household recycled?	In the last year, approximately what percentage of plastic bottles/containers has your household recycled?
D20	In your opinion, how responsible are each of the following actors for reducing the use of single-use plastic?<br>• Government institutions<br>• Companies in all sectors (deciding which packaging to place on the market)<br>• Consumers (through their choices)	In your opinion, how responsible are each of the following actors for reducing the use of single-use plastic?<br>• Government institutions<br>• Companies in all sectors (deciding which packaging to place on the market)<br>• Consumers (through their choices)
D21	How much do you agree with the following statement? "It is necessary to adopt measures to reduce the use of single-use plastic items (e.g., shopping bags, straws…)."	How much do you agree with the following statement? "It is necessary to adopt measures to reduce the use of single-use plastic items (e.g., shopping bags, straws…)."

Items that were modified in response to citizen input are highlighted in bold
